# Systematic Enzyme Mapping of Cellular Metabolism by Phasor-Analyzed Label-Free NAD(P)H Fluorescence Lifetime Imaging

**DOI:** 10.3390/ijms20225565

**Published:** 2019-11-07

**Authors:** Ruth Leben, Markus Köhler, Helena Radbruch, Anja E. Hauser, Raluca A. Niesner

**Affiliations:** 1 Biophysical Analytics, Deutsches Rheuma-Forschungszentrum (DRFZ), 10117 Berlin, Germany; markus.koehler@drfz.de; 2 Dynamic and Functional in vivo Imaging, Freie Universität Berlin, 14163 Berlin, Germany; 3 Institute for Neuropathology, Charité–Universitätsmedizin Berlin, 10117 Berlin, Germany; helena.radbruch@charite.de; 4 Immune Dynamics, Deutsches Rheuma-Forschungszentrum (DRFZ), 10117 Berlin, Germany; hauser@drfz.de; 5 Immunodynamics and Intravital Microscopy, Charité–Universitätsmedizin Berlin, 10117 Berlin, Germany

**Keywords:** NADH and NADPH fluorescence lifetime imaging, enzyme activity, 3T3-L1 cell line, phasor approach, cellular metabolism

## Abstract

In the past years, cellular metabolism of the immune system experienced a revival, as it has become clear that it is not merely responsible for the cellular energy supply, but also impacts on many signaling pathways and, thus, on diverse cellular functions. Label-free fluorescence lifetime imaging of the ubiquitous coenzymes NADH and NADPH (NAD(P)H-FLIM) makes it possible to monitor cellular metabolism in living cells and tissues and has already been applied to study metabolic changes both under physiologic and pathologic conditions. However, due to the complex distribution of NAD(P)H-dependent enzymes in cells, whose distribution continuously changes over time, a thorough interpretation of NAD(P)H-FLIM results, in particular, resolving the contribution of various enzymes to the overall metabolic activity, remains challenging. We developed a systematic framework based on angle similarities of the phase vectors and their length to analyze NAD(P)H-FLIM data of cells and tissues based on a generally valid reference system of highly abundant NAD(P)H-dependent enzymes in cells. By using our analysis framework, we retrieve information not only about the overall metabolic activity, i.e., the fraction of free to enzyme-bound NAD(P)H, but also identified the enzymes predominantly active within the sample at a certain time point with subcellular resolution. We verified the performance of the approach by applying NAD(P)H-FLIM on a stromal-like cell line and identified a different group of enzymes that were active in the cell nuclei as compared to the cytoplasm. As the systematic phasor-based analysis framework of label-free NAD(P)H-FLIM can be applied both in vitro and in vivo, it retains the unique power to enable dynamic enzyme-based metabolic investigations, at subcellular resolution, in genuine environments.

## 1. Introduction

The importance of cellular metabolism in immunology has become increasingly recognized in the last years. Although, in the last decades, molecular immunology mainly focused on signaling pathways within various immune cell subtypes and their supporting cells, such as stroma cells, it has become evident that cellular metabolism does not merely provide energy necessary for transcription and biosynthesis in immune cells, but is tightly interconnected with their function [1,2]. For instance, the process of selection and further differentiation of germinal B cells was shown to be directly linked to cellular metabolism [3]. These correlations between metabolism and immune cell function and dysfunction still require extensive investigations, as oxygen consumption measurements related to enzymatic activity are mainly performed ex vivo and not at the single-cell level. Only by using synthetic probes, such as dichlortris(1,10-phenanthroline) ruthenium(II) hydrate (Ru(Phen)) [4], the oxygen concentration in cells and tissues can be measured at the level of single cells [5]. However, these approaches allow only an indirect link to enzymatic (metabolic) activity.

Fluorescence lifetime imaging (FLIM) was developed three decades ago, and since then it has been applied to study cellular function in a quantitative manner [6]. FLIM generates images in which contrast is obtained by the excited-state lifetime τ of fluorophores instead of their intensity, therefore, having negligible experimental bias. The combination of FLIM with two-photon microscopy—the technology of choice when performing dynamic deep tissue imaging in living mammals [7]—allowed it to reach its full potential under genuine conditions and to study tissue and cellular function and dysfunction in vivo. In this way, using FLIM, molecular mechanisms underlying neuronal damage and oxidative stress have been understood in chronic neuroinflammation [8,9,10,11,12], neurodegeneration [13], and glioblastoma [14,15]. Furthermore, the shift from differentiation towards uncontrolled proliferation in cancer cells has been elucidated in several primary and metastatic tumor cells [16], and the physiologic process of differentiation has been unraveled in cells and organs [17,18,19,20,21].

A multitude of approaches have been developed over the years to measure fluorescence lifetimes in bulk solution, without spatial resolution. Later on, they have been applied to microscopy to perform FLIM. Both frequency-domain and time-domain methods have been developed to meet different needs in biosciences, biomedicine and medicine [22,23,24,25,26,27,28]. Frequency-domain technologies are particularly adequate for fast, high-throughput measurements [22,29,30], as well as for retrieving multiple differing fluorescence lifetimes from complex samples using several modulation frequencies, ranging from kHz to MHz [31,32]. Among the time-domain technologies, time-correlated single-photon counting (TCSPC), which requires pulsed excitation as delivered by two-photon microcopy, is the method of choice to comprehensively acquire the molecular complexity within living cells in deep tissue, despite its rather slow speed (1–10 s/frame) [11,33]. TCSPC directly measures the fluorescence decay of all contained fluorophores; however, its thorough analysis remains a challenge especially in complex microenvironments as found in cells.

The fluorophores first imaged by FLIM were the coenzymes nicotinamide adenine dinucleotide—NADH and nicotinamide adenine dinucleotide phosphate—NADPH [6] (hereafter NAD(P)H), i.e., ubiquitous coenzymes governing energy production in all cells across species, as well as being responsible for various biosynthetic processes and influencing diverse signaling pathways [34]. NAD(P)H-FLIM [35] has been extensively employed in cancer research, revealing shorter NAD(P)H fluorescence lifetimes in tumor cells as compared to controls, presumably due to a shift from oxidative phosphorylation (OxPhos) towards glycolysis [16,36,37,38,39,40,41]. By enabling the differentiation between the two major cellular metabolic pathways, namely, glycolysis and OxPhos, it has also revealed the tight link between metabolism and differentiation in other cell types, for example, in neuronal stem cells [18], in mesenchymal stromal cells during their differentiation towards various fates such as adipocytes, in chondrocytes, or osteoblasts [21,42,43,44], and in enterocytes in the small intestine [17]. Additionally, by dynamically monitoring the activation of NADPH oxidases, this technique has been applied to monitor the oxidative burst and massive oxidative stress production during infection in the small intestine [45], as well as apoptosis in cells [46].

Both fluorescence lifetime spectroscopy and FLIM are able to differentiate between the states of free and enzyme-bound coenzymes NADH and NADPH. In the free state (unbound to enzymes), the fluorescence of the adenine is intramolecularly quenched by nicotinamide, resulting in a short fluorescence lifetime of around 450 ps [29,47]. Upon enzymatic binding, this quenching is prevented due to steric hindrance caused by the specific binding site of the coenzyme, resulting in enzyme-specific prolongation of the fluorescence lifetime towards 2000 ps [48]. Additionally, the fluorescence lifetime of both free and enzyme-bound NAD(P)H, as with the lifetimes of the majority of fluorophores, is also influenced by other factors such as refractive index [49], solvent polarity [29], pH [50,51], ion concentration [52], or the degree of freedom for the diffusional rotation of the fluorescent molecules. Gregorio Weber in 1970, and then Joseph R. Lakowicz in 1992, showed that there are differences in the fluorescence lifetime of NADH and NADPH depending on the binding site of the enzyme [6,29]. This principle has been used for a number of isolated enzymes. However, in living cells, multiple pathways employing NAD(P)H-dependent enzymes are active at any given time, making it hard to interpret NAD(P)H-FLIM data derived from living cells with respect to the proportionate usage of certain enzymatic reactions. No framework allowing a systematic interpretation of the NAD(P)H-FLIM data with respect to both metabolic activity and specific, predominant enzymatic activity is available yet. As such, there are apparently contradictory interpretations, indicating that the fluorescence lifetime of NAD(P)H bound to enzymes depends on the coenzyme alone [53] is caused by other molecular species, e.g., oxidized lipids [54], or has a fixed value of ~2000 ps [23,39,48,55,56] or ~3400 ps [54,57].

Here, we established a general framework retrieving two kinds of information from NAD(P)H-FLIM data: (i) the fraction of free vs. enzyme-bound NAD(P)H present in cells, i.e., a measure of immediate metabolic activity, and (ii) which enzymes are the major contributors to the NAD(P)H-dependent metabolic activity in a certain cellular compartment, at a particular time point. Our approach is based on the measurement of the fluorescence lifetime of NADH and NADPH free and bound to enzymes, which are highly and ubiquitously expressed by mammalian cells. We performed the measurements of homogeneous mixtures of NAD(P)H and single enzymes in buffered media resembling the pH, ion concentration, solvent polarity, and refractive index of cells to ensure that only the binding state of the coenzymes affected the fluorescence lifetime. Typical images acquired by NAD(P)H-FLIM on cells and tissues have a voxel size of 500 nm × 500 nm × 1500 nm. Considering the mean concentration of proteins in a cell [58], we calculated that a number in the range 10^8^–10^9^ protein molecules are present within a voxel. Of these proteins, 18% are metabolic enzymes [58]. Even if the number of enzyme molecules may vary between different cell populations, a large number of enzymes are competing to bind NAD(P)H. Therefore, we expect that only the activity of highly abundant NAD(P)H-dependent enzymes will have a major impact on the NAD(P)H fluorescence lifetime. Unpublished RNA-Seq data from our colleagues showed that out of 191 NAD(P)H-dependent enzymes expressed by mesenchymal stromal cells, 16 ubiquitously expressed metabolic enzymes, their including isoforms are contained within the 50 most abundantly expressed genes. These findings are in line with other studies, i.e., cancer cells [58], and presumably hold true for many other cell types. From these highly expressed metabolic enzymes we only excluded complex I, due to difficulties in maintaining the activity of its flavin mononucleotide-binding domain in solution. The NADH-binding unit of complex I is one of the less abundant among these 16 enzymes. We focused on these highly abundant NAD(P)H-dependent metabolic enzymes (covering the metabolic enzymes but excluding their isoforms), i.e., malate dehydrogenase (MDH), lactate dehydrogenase (LDH), glyceraldehyde-3-phosphate dehydrogenase (GAPDH), glucose-6-phosphate dehydrogenase (G6PDH), pyruvate dehydrogenase (PDH), alcohol dehydrogenase (ADH), C-terminal binding protein 1 (CTBP1) [57], and isocitrate dehydrogenase (IDH); complemented by the still abundant enzymes, i.e., hydroxyacyl-coenzyme-A dehydrogenase (HADH) and sorbitol dehydrogenase (SDH); and enzymes playing distinct roles in the cells, i.e., inducible nitric oxide synthase (iNOS) and the NADPH oxidases family (NOX1-4 and DUOX1,2) [59], which play a major role in the catalysis of oxidative burst. As the quaternary structure of the NAD(P)H binding site and the related catalytic function in these NAD(P)H-dependent enzymes is highly conserved across species and in various cellular organelles, it was sufficient to investigate only one variant for each enzyme.

Our framework to evaluate NAD(P)H-FLIM data by the phasor approach, i.e., a model-free analysis tool of FLIM data [59,60], additionally uses for normalization the signal-to-noise ratio of the NAD(P)H intensity image for signal quality control. It decouples the metabolic activity level, i.e., the fraction of free to enzyme-bound NAD(P)H, from the type of predominant enzymatic activity, as indicated by the angle similarity to the phase vector of NAD(P)H bound to pure enzymes. We validated the performance of our analysis framework on homogeneous mixtures of NADH and enzymes as well as on a stromal-like 3T3-L1 cell line. Our systematic framework meets the increasing demand to interpret in a reliable manner, the metabolism of immune and stromal cells, at subcellular resolution, by NAD(P)H-FLIM, both in cell culture [61] and in living tissues and organisms [8,9,13].

## 2. Results

### 2.1. Benchmarking of NAD(P)H-FLIM Data Evaluated Using the Phasor Approach

To define the image and signal properties to ensure reliable data interpretation, we performed NADH-FLIM measurements in standardized NADH solutions of various concentrations, as well as in NADH mixtures with metabolic enzymes, i.e., malate dehydrogenase (MDH) or lactate dehydrogenase (LDH).

**Figure 1:**
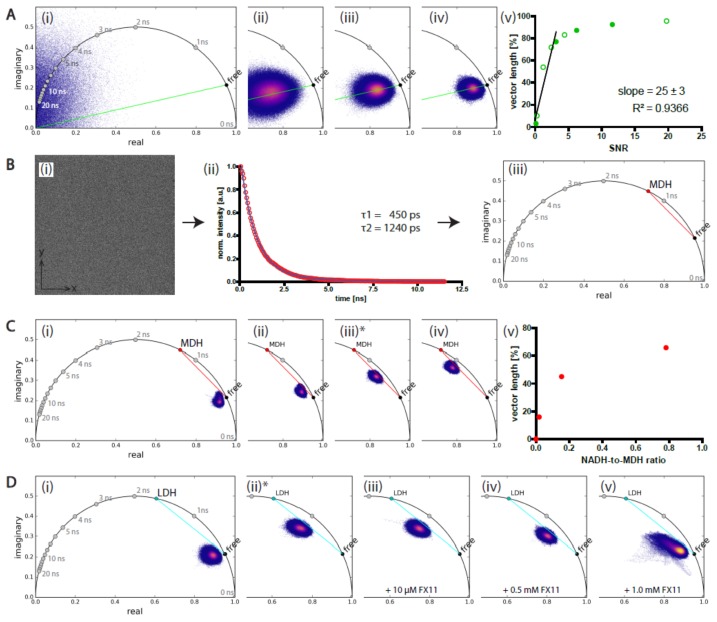
Benchmarking of NAD(P)H-FLIM data evaluated by the phasor approach. Phasor plots of mixtures of NADH and various enzymes solved. Gray dots onto half circle show the positions of 0 ns ≤ τ ≤ 20 ns in 1 ns-steps in phase domain. Phasor cloud: violet to orange–yellow false color representation of 3D histogram of phasor distribution; violet: low frequency; orange to yellow: increasing frequency, each dot in phasor plot has a counterpart in the xy fluorescence lifetime image. (*n* = 3 measurements in the same sample). (**A**) NADH solved in MOPS medium: (**Ai**) only nonfluorescent MOPS medium (background BG), (**Aii**) 5 µM, (**Aiii**) 10 µM, and (**Aiv**) 20 µM NADH. Green line indicates the phase vector pointing towards the position of free NADH onto the half circle. (**Av**) Graph displays signal–noise ratio (SNR) versus vector length in percent (as described in Results). The increase in SNR is caused by an increasing NADH concentration at constant excitation power. Filled circles indicates the data shown in panels (**Ai**–**iv**), blank circles represent results of the same experiments with other NADH solution concentrations. The line describes the linear regression of the displayed data points. (**B**) Strategy to determine the position of the phase vector of NAD(P)H bound to pure enzymes onto the half circle; here, 50 µM NADH and 39.1 µM MDH solved in MOPS medium: (**Bi**) homogeneous intensity image 512 × 512 pixel covering 200 µm × 200 µm, (**Bii**) averaged time domain decay curve over all pixel with biexponential fit, as line and data points (red circles)., (**Biii**) τ_2_ was transferred to phase domain to mark MDH’s position onto the half circle (red dot), red vector connects position of free NADH and MDH bound to NADH. (**C**) Mixtures of NADH and MDH solved in MOPS medium: (**Ci)** only 200 µM NADH, (**Cii**) 132 µM NADH and 2.6 µM MDH [ratio MDH:NADH 0.019], (**Ciii**) 50 µM NADH and 7.7 µM MDH [0.154], and (**Civ**) 50 µM NADH and 39.1 µM MDH [0.782]. The position of NADH bound to MDH fluorescence lifetime was determinate as described in (**B**). (**Cv**) Graph displays MDH:NADH ratio vs. vector length [%]. Red dots indicate the data shown in (**Ci-iv**). (**D**) NADH, LDH, and FX11 solved in MOPS solution: (**Di**) only 50 µM NADH; (**Dii**) 50 µM NADH and 10 µM LDH; (**Diii**) 50 µM NADH, 10 µM LDH, and 10 µM FX11; (**Div**) 50 µM NADH, 10 µM LDH, and 0.5 mM FX11; and (**Dv**) 50 µM NADH, 10 µM LDH, and 1.0 mM FX11. The position of LDH lifetime was determined as described in (**B**) (τ_2_ = 1600 ps). After adding the LDH inhibitor, FX11, the experimental data cloud migrates back to free NADH with increasing FX11 concentration. The asterisks indicate the data sets used to calculate the SNR (Figure S3) as well as calculate τ_2_ to mark the half circle. The cyan lines represent the trajectories on which the data cloud migrates if the enzyme activity increases.

We evaluated the FLIM data acquired in time-domain by time-correlated single-photon counting in a two-photon microscope using the phasor approach. The raw data consist of a fluorescence decay curve of the fluorophore, in our case NADH, in each pixel of the image. The decay curve is monoexponential if all fluorophore molecules in the volume corresponding to a pixel in the image have the same molecular environment. If the molecules have various molecular environments, the decay curve became multiexponential. The characterizing parameter of the fluorescence decay curve is the fluorescence lifetime, i.e., the time period the fluorescence intensity decreases to 1/*e* (where *e* is Euler’s number) of its maximum value immediately after the excitation pulse. In the phasor plot, the lifetimes follow a naturally logarithmic distribution on the half-circle (Figure 1(Ai)).

#### 2.1.1. Low Signal-to-Noise Ratio of the Image Affects the NAD(P)H-FLIM Data

Both mono- and multiexponential decays are convolved with shut noise, i.e., an undamped multifrequency oscillation, which is also transformed to the phase domain and lies within the area defined by the half circle. To quantify the role of shut noise, we first investigated the impact of the signal-to-noise ratio of the image on the results of the phasor-evaluated NAD(P)H-FLIM data by varying either the concentration of NADH while the laser power stayed the same at 100 mW (Figure 1A) or by varying the excitation laser power while the NADH concentration was fixed at 50 µM (Video S1 and Figure S1). 

The signal-to-noise ratio (SNR) is defined as the ratio of the mean background-free fluorescence signal (*µ*_signal_ − *µ*_BG_) and the noise of the background (shut noise), i.e., the standard deviation of the background intensity distribution (σ_BG_):

(1) $$SNR=\frac{\mu_{\mathrm{siginal}}-\mu_{\mathrm{BG}}}{\sigma_{\mathrm{BG}}}$$

An increasing NADH concentration results in increasing signal-to-noise-ratio (SNR), identifiable by decreasing width of the phasor cloud, i.e., the distribution of the phase vectors measured in each pixel of the image, in the phasor plots. Figure 1(Av) shows the SNR vs. the vector length, which is the ratio of the vector “(0|0) to free NAD(P)H position onto the half circle” (green line in Figure 1(Aii–v)) and the vector connecting (0|0) with the center of the phasor cloud, in percent. Increasing the laser power while the NADH concentration is fixed shows the same effect.

Independently of the way we modified the SNR in the image, we found that for SNR values above 5, the quality of the fluorescence signal delivered results very similar to those predicted by the theory of the phasor approach. No experimental data are able to reach the accuracy of theoretically calculated values due to the shut noise contained therein. In the range of SNR values less than 5, the vector length, i.e., *Re*² + *Im*² (where *Re* is the real part and *Im* the imaginary part of the complex number), decreases linearly with decreasing SNR, whereas the direction of the vector given by the angle to the abscise remains constant.

#### 2.1.2. Validation of Phasor-Evaluated FLIM Data on Mixtures of NADH and MDH or LDH

The theory of the phasor approach predicts that, for a mixture of fluorophore molecules experiencing two different molecular environments, the vector will point onto the segment connecting the positions on the half circle corresponding to fluorophore molecules experiencing only one of these molecular environments. Therefore, the phase vectors of a FLIM measurement of a mixture of NADH and malate dehydrogenase (MDH) point onto the segment connecting the position of free NADH onto the half circle and that of NADH completely bound to MDH.

As the NADH:MDH mixture was a homogeneous solution containing only two fluorescent species, the time domain decay curve was averaged over all pixels in order to smooth it for proper biexponential fitting (least-square sum method). This fit results in τ_1_ = 450 ps and τ_2_ = 1240 ps (*R*² = 0.99956). τ_1_ is the lifetime of free NADH and is in good agreement to known data [47]. τ_2_ indicates the lifetime of NADH bound to MDH and was transferred to the phase domain to mark the position of MDH onto the half circle (Figure 1(Bi–iii)).

In Figure 1(Ci–iv), we investigated mixtures of NADH and MDH with various relative concentrations of coenzyme and enzyme given by the ratio MDH:NADH. We performed the FLIM experiments in such a way that the SNR remained the same for all measurements. SNR was calculated for panel (iii) (marked by asterisk) and was 39.6 (Figure S3). As expected, the position of the phasor cloud migrates from the free NADH position, towards the position of NADH fully bound to MDH with increasing MDH:NADH ratio, along the connecting vector. FLIM, especially when transformed to phase domain, is very sensitive to noise. Noise has an infinite fluorescence lifetime, which is positioned around the origin (0|0) in the phasor plot. Although our SNR is very high, in the case of MDH and LDH, respectively, noise still represents a third “lifetime component”. For this reason, the phasor cloud does not lie exactly on the connecting line, but migrates slightly parallel to it. In the following analysis (especially later in the enzyme assignment), this circumstance was taken into account by the fact that the vector free-enzyme starts at the center of the measured phasor cloud and does not start from the marked position on the half circle (Figure 3A). 

Figure 1(Cv) shows the MDH:NADH ratio vs. the vector length, which is the ratio of the vector “free NADH to the center of the center of the phasor cloud” to “free NADH to NADH fully bound to MDH position on the half circle” (red line), in percent.

NADH-FLIM experiments on mixtures of NADH and lactate dehydrogenase (LDH) performed at a SNR far above 5 generally revealed the same valid behavior. Due to the low LDH concentration (10 µM, LDH:NADH ratio = 0.2), the phasor cloud did not reach the position corresponding to NADH fully bound to LDH. By adding the LDH inhibitor FX11 at various concentrations, we could reverse the binding of NADH to LDH, which resulted into a shift back to free NADH in the phasor plot (Figure 1D and Figure S2). The smearing of the experimental data at high FX11 concentrations in panel (Figure 1(Dv)) results from enzyme denaturation since the intensity image becomes in this case heterogeneous. The SNR remains constant at 22.3 (Figure S3).

### 2.2. Enzyme-Based Reference System to Interpret Label-Free NAD(P)H-FLIM Data in Cells

**Figure 2:**
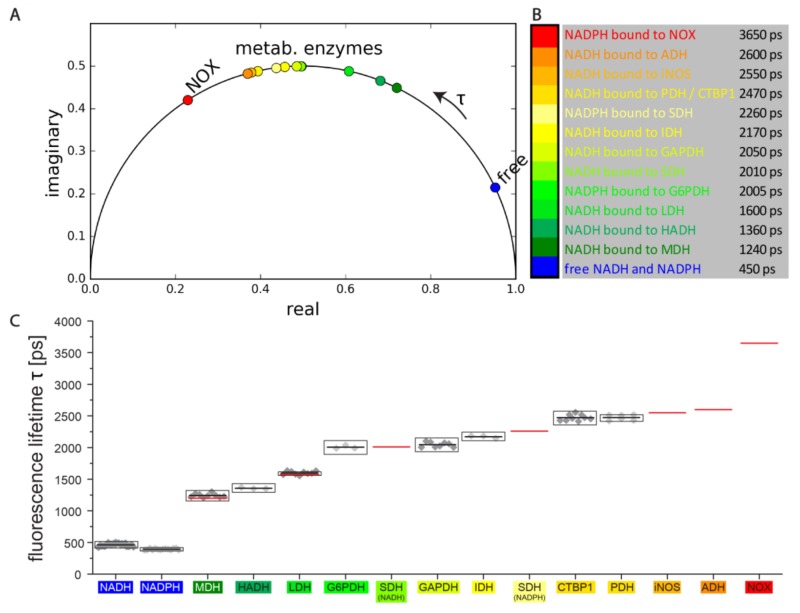
Reference system of NADH and NADPH fluorescence lifetimes and phase vectors in the free and enzyme-bound state. (**A**) Phasor plot with the positions of the coenzymes NADH and NADPH fully bound to the identified relevant, abundant NAD(P)H-dependent enzymes, as chosen by from RNA sequencing data. The positions on the plot have been either determined as described for NADH and MDH in Figure 1B from our experimental FLIM data on mixtures of coenzyme and pure enzyme or calculated from our previously published data on such mixtures. The arrow outside the half-circle shows the direction in which the fluorescence lifetime τ increases. (**B**) List of measured fluorescence lifetimes τ_2_ of NADH or NADPH fully bund to enzymes. (**C**) The fluorescence lifetimes in panel (B) were determined as described in Figure 1B, fluorescence lifetimes calculated from single images represented as gray dots, average values as black lines, standard error of the mean (s.e.m.) values over all pixels in all measured images per coenzyme/enzyme mixture as black boxes. The list was completed by our previously measured fluorescence lifetimes [6] measured for NADH and NADPH bound to SDH, NADH bound to iNOS, NADH bound to ADH, and NADPH bound to enzymes of the NOX family (red lines). For LDH and MDH the literature data of other groups are indicated as red lines. The color coding in panels (A–C) is consistent.

The basic concept of our approach for interpreting NAD(P)H-FLIM data takes into account the most likely states in which NADH and NADPH can be found in cells and tissues. Except for the free state, we find the coenzymes bound to diverse enzymes, in a mixture containing ~350 NADH- and 300 NADPH-dependent enzymes. However, many of these enzymes are so rare that they have a negligible effect on the overall fluorescence decay of the coenzymes. We identified from RNA-Seq data the most abundant and, thus, relevant NADH and NADPH-dependent enzymes and completed the list with NADPH oxidases, i.e., enzymes responsible for oxidative burst generation in certain cell types. The phasors of free NAD(P)H and fully bound NAD(P)H to these enzymes, respectively, are depicted in Figure 2A. The corresponding fluorescence lifetimes are listed in Figure 2B. Using FLIM, we measured mixtures of NADH or NADPH and several of these pure enzymes (Figure 2C) and evaluated the data using the phasor approach as described in Figure 1B for the NADH/MDH mixtures. The list is completed by our previously published data on other relevant enzymes measured extracellularly in solution [8]. The only exception represents the NADPH Oxidases family (here NOX), which has a fluorescence lifetime and position in the phasor plot and was determined intracellularly using chemical inhibition, activation, and knockout strategies [33,55]. We paid particular attention to perform all FLIM measurements on NAD(P)H solutions and NAD(P)H-enzyme mixtures in buffered media resembling similar pH, ion concentrations, and refractive index as the cellular environment to avoid lifetime artifacts caused by these parameters. As shown by the good agreement of our results of NADH/MDH and NADH/LDH mixtures with the findings of other groups [47] (red lines in Figure 2C), our reference system is a generally valid system that can be applied to any cell or tissue type. The fact that we were not able to measure NADH bound to functional complex I, neither extracellularly nor intracellularly, is a limitation of our reference system. 

### 2.3. Analysis Framework of NAD(P)H-FLIM Data: Validation on Homogeneous NAD(P)H-Enzyme Mixtures

**Figure 3:**
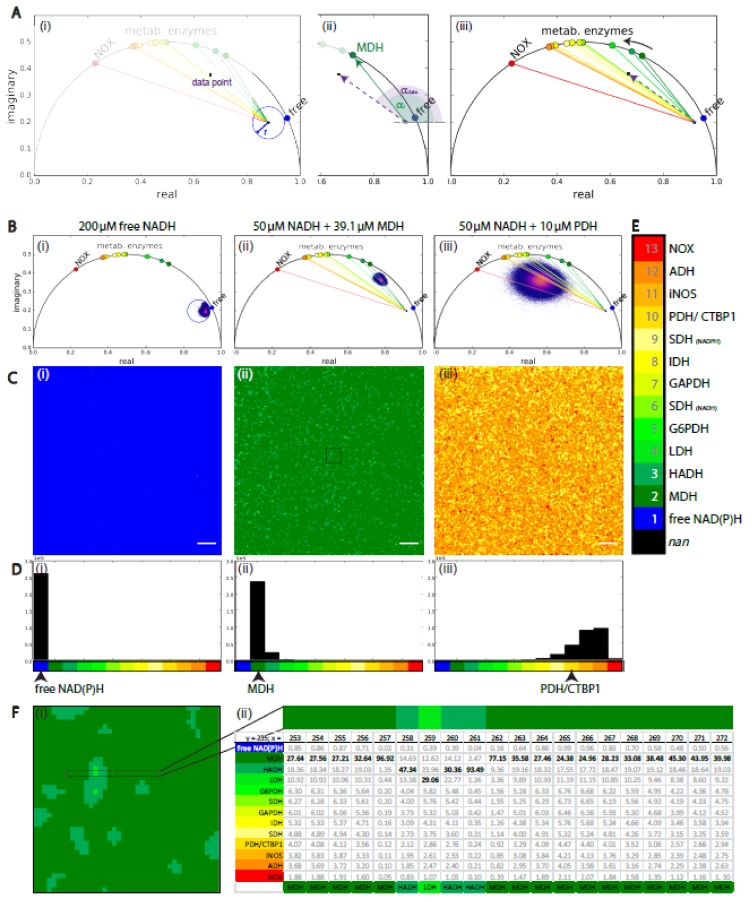
Validating the systematic framework of NAD(P)H-FLIM data analysis. (**A**) Strategy for assigning a data point (violet) to free or enzyme-bound NAD(P)H. (**Ai**) Inspection whether the data point is within the radius *r*, which indicates the measured phasor position of free NAD(P)H. The blue circle describes the experimental area of only free (unbound) NAD(P)H. (**Aii**) If (**Ai**) does not apply, the angles α_data_ and α_i_ (*i* = enzyme index) are calculated and compared for every single NAD(P)H-dependent enzyme by equations (Equations (2) and (5)). The data point is assigned to the enzyme for which (Equation (5)) it becomes maximal. The purple dotted arrow describes an experimental trajectory vector of a pixel, whereas the solid green arrow stands for the expected trajectory vector of NADH bound only to MDH (enzyme index 2). The green sector describes the angle α_2_ between the x-axis (Re-axis) and the green trajectory vector whereas the purple sector describes the same angle α_data_ calculated from the experimental data (purple trajectory vector). (**Aiii**) Phasor plot containing all enzymes used for benchmarking and an experimental trajectory vector of a pixel (purple dotted arrow). The enzymes are indexed from 2 to 13. 1 corresponds to free NAD(P)H. These numbers are associated with specific colors as displayed in (**E**). The colors of the dots in the phasor plot (**Aiii**) correspond to expected phasor points of NADH or NADPH entirely bound to the enzymes indexed from 2 through 13. The colored lines correspond to the trajectory vectors of the same enzymes, color-encoded as displayed in (**E**). (**B**) Phasor plots of NADH mixed with different enzymes solved in MOPS medium. (**Bi**) 200 µM free NADH, blue circle indicates free NAD(P)H (**Bii**) 50 µM NADH and 39.1 µM MDH (same data as shown in Figure 1(Civ)), (**Biii**) 50 µM NADH and 10 µM PDH. (**Ci**–**iii**) to (**Bi**–**iii**) corresponding enzyme maps, the enzymes were assigned as described in (**A**). (**Di**–**iii**) to (**Ci**–**iii**) corresponding histograms. Scale bar corresponds to 20 µm. (**E**) Color legend of enzyme assignment. The gray numbers represent the enzyme indices used throughout this work. *nan* = “not a number“: a non-numerical place holder in the enzyme matrix (e.g., after thresholding) used to represent the enzyme map spatially correct. All other abbreviations refer to the enzymes mentioned in the introduction. (**F**) (**Fi**) Magnified region of interest form (**C**) (**Fii**) representing the enzyme assignment. ROI is marked in (**Cii**) by a black square. (**Fii**) 20 × 1 pixel line (ROI marked in (**Fi**)), in (**Cii**) pixel line y = 235, 253 ≤ x ≤ 272. The table in (**Fii**) displays the normalized angle similarities *w_i_* (Equation (5)) for all enzymes of the reference system in each pixel of the selected ROI. Maximum values are displayed bold. The lowest line of the table shows the enzyme assignment based on the maximum *w_i_* value. The pixel line in (**Fii**) is 7.8 µm long.

Based on the previously generated reference system of coenzymes bound to pure enzymes, we created a systematic analysis framework of NAD(P)H-FLIM data. First, we determined the SNR of the NAD(P)H fluorescence intensity image, which is a quality criterion of the time-domain signal. After calculating the phasor in each pixel of the image, we selected the data points that were located within the circular area in the phasor plot identified as “free NAD(P)H” area (Figure 3(Ai)). The radius *r* and position of this circular area was determined from the measured distribution of free NADH (Figure S4). We attribute those data points to “free NAD(P)H” and consider that a cell would not show any metabolic activity in those image areas where these data points originate from. To assign the data point to enzymes of our reference system, we calculated the angles α_i_ and α_data_ (Figure 3(Aii)) in the remaining pixels. α_i_ is the angle of the vector connecting the position of measured free NAD(P)H and the positions of NAD(P)H fully bound to the pure enzyme of our reference system with the index *i* (e.g., *i* = 2 for MDH, represented as dark green, solid line), and α_data_ is the angle of the vector connecting the position measured free NAD(P)H and each experimental data point (violet, dashed line) (Figure 3(Aii,Aiii)). Furthermore, we calculated the absolute value (expressed by $\left|\ldots \right|$) of angle similarity *q_i_* to each enzyme of the reference system as follows,

(2) $$q_{i}=\left|\frac{\alpha_{\mathrm{data}}}{\alpha_{i}}-1\right|$$

Further, we normalized the angle similarities *q_i_* for all enzymes in each pixel and obtained the weights *w_i_* of the angle similarities *q_i_* as follows,

(3) $$w_{i}q_{i}=const$$

(4) $$\sum w_{i}=1$$

(5) $$w_{i}=\frac{1}{q_{i}\sum \frac{1}{q_{i}}}$$

Equation (5) gives the assignment probability for each enzyme of our reference system. After multiplying by 100, it may reach from 0 to 100 and the data point is assigned to the enzyme for which this value reaches its maximum. Repeating this procedure for each pixel of an image generated an enzyme map as shown in (Figure 3(Ci–iii)). At the same time, all weights *w_i_* may be retrieved as a 3D matrix (2D × 13), as depicted in Figure 3(Fii), for an exemplary 20 × 1 pixel line. In the case that the maximum weight *w_i_*, on the basis of which the enzyme assignment is performed, is similar to the neighboring enzymes, the comparison of the weights in the 3D matrix becomes relevant for data interpretation.

In Figure 3B–D, we validated our analysis framework on homogeneous mixtures of free NADH (i), NADH and MDH (ii), and of NADH and PDH (iii), respectively. The enzyme maps (Figure 3C) of free NADH, as well as NADH mixed with MDH, show a high accuracy of the analysis framework, substantiated by the histograms shown in Figure 3D. The histograms show that 99.9% of the pixels are correctly assigned to free NADH (Figure 3(B,Ci)) and 97.7% of the pixels in Figure 3(B,Cii) are correctly assigned to MDH (enzyme index = 2). The SNR of free NADH image was 39.6 and for MDH:NADH mixture image 22.3. In contrast, the histograms of the enzyme map of NADH mixed with PDH showed that 37.4% of the data points were assigned to ADH, 35.1% to iNOS and only 17.4% to PDH/CTBP1. This is partially due to the high similarity of the lifetimes of NADH if bound to PDH/CTBP1, iNOS, and ADH (see Figure 2C), which makes an accurate assignment difficult. In such a case, the expression level of these enzymes and their subcellular distribution need to be taken into account. Furthermore, the SNR in the PDH:NADH mixture image was with a value of 9.99 lower than that for free NADH and MDH:NADH mixture. As becoming evident from the broad phasor cloud in the corresponding phasor plot (Figure 3(Biii)), in a pixel-based evaluation the SNR criterion needs to be set higher for a sufficient accuracy.

**Figure 4:**
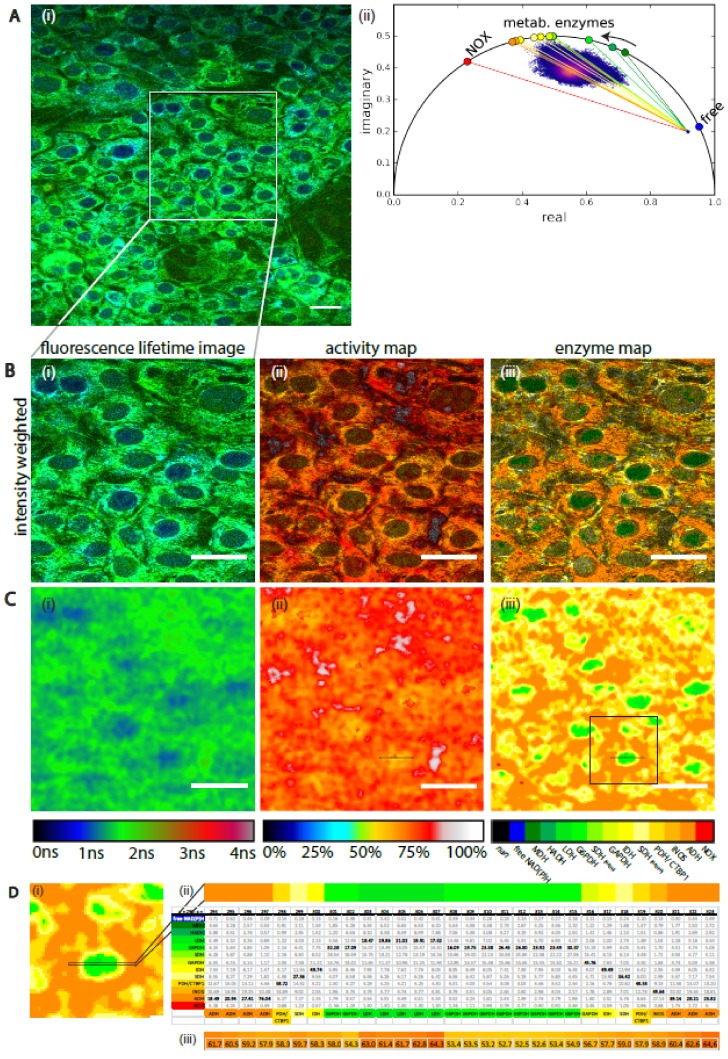
Label-free NAD(P)H-FLIM reveals subcellular heterogeneity of enzyme activity but homogeneous metabolic activity in stromal-like 3T3-L1 cells. (**A**) Spatial-resolved fluorescence lifetime image of living 3T3-L1 cells. (**Ai**) Intensity-weighted fluorescence lifetime image 505 × 505 pixel covering 500 µm × 500 µm and (**Aii**) corresponding phasor plot. (**B**,**C**) 198 µm × 198 µm region of interest, (B) intensity weighted, (**C**) unweighted images of (**Ci**) fluorescence lifetime image, (**Cii**) distance map, and (**Ciii**) enzyme map; white bar indicates 50 µm. The “distance map” maps the ratio in percent of the vector “free NADH to data point” to “free NADH to assigned enzyme position on the half circle”. The mean vector length is (68 ± 6) %. (**D**) Magnified region of interest form (**Ciii**), the ROI is marked by black square and asterisk, (**Dii**) 30 × 1 pixel line (ROI marked in (**Di**) in the original image it is the pixel line y = 290, 294 ≤ x ≤ 323) and table with corresponding *w_i_* of Equation (5) for each shown pixel and each enzyme. Maximum values are highlighted in black. The lowest line of the table shows the enzyme assignation by finding the maximum, (**Diii**) the corresponding vector length in that pixel line and is also marked in (**Dii**). The pixel line in (**Dii**) and (**Diii**) is the magnified picture of the small rectangle displayed in (**Cii**) and in (**Ciii**). The dimension of the pixel line is 30 µm.

The island-shaped structures in the enzyme maps, especially in the case of the mixture NADH and PDH, do not result from a badly solved enzyme solution, but rather originates from the Gaussian blurring of the time-resolved fluorescence data; one of the very first steps of the analysis. By spatially blurring the time domain raw data, the time resolution is improved at the cost of spatial information. The Gaussian blur with a radius of two pixel (σ = 2) is a good compromise between time and space accuracy (Figure S5). In the case of homogeneous solutions, the spatial resolution is irrelevant, but this becomes important when performing label-free NAD(P)H-FLIM of cells and tissues (Figure 4). In this case, we additionally mapped the ratio length of the vectors we used to calculate angle α_i_ and α_data_ as defined above. This ratio represents the fraction of free NAD(P)H vs. bound NAD(P)H to the assigned enzyme in each pixel of the image, and thus is a hint for the metabolic activity in this area of the cell.

### 2.4. Validation of the NAD(P)H-FLIM Analysis Framework on Stromal-Like 3T3-L1 Cells

We applied our systematic analysis framework on NAD(P)H-FLIM data acquired in 3T3-L1 cells (Figure 4A)—a cell line which has mesenchymal stromal cell-like properties and can differentiate into adipocytes when appropriately stimulated. In the bone marrow, stromal cells, the heterogeneity of which is still poorly understood, are known to fulfill various functions. Particularly, in the deep marrow cavity of long bones, they, together with the vasculature, form crucial components of the survival niche for various immune cell populations such as long-lived plasma cells [61], as well as for hematopoietic stem cells [62]. Both in homeostasis and during bone regeneration, stromal cells may differentiate towards osteoblasts, chondrocytes or adipocytes. In different phases of life, the balance between these differentiation pathways and the nondifferentiated state is expected to shift, e.g., in aged individuals, or due to metabolic syndrome, differentiation towards adipocytes is more dominant [63]. The various differentiation pathways have been previously linked to differences in the NAD(P)H metabolism as measured by FLIM [20,42,47].

In particular, differences between the mean NAD(P)H fluorescence lifetime in the nucleus vs. cytoplasm have been reported. However, it is not yet clear which metabolic mechanisms underline these observations.

The SNR in the image ranged between 28 ± 6 in nuclei and 48 ± 16 in cytoplasm, thus our measurements lies within a reliable range. Note that these SNR values could be only reached by summing up subsequently acquired five time-domain images of the cells. The NAD(P)H fluorescence signal is dim in cells and an increase in excitation power would entail the risk of photodamage [64]. Our systematic framework for NAD(P)H-FLIM in 3T3-L1 cells revealed similar metabolic activity as substantiated by the distance maps in Figure 4(B,Cii), i.e., similar fraction of free NAD(P)H. Although there was a certain degree of heterogeneity among the metabolic activity, we observed no significant differences between cytoplasm and nucleus in this respect. In contrast, we found, in the enzyme map, predominant activity of different NAD(P)H-dependent enzymes in nuclei as compared to the surrounding cytoplasm (Figure 4(B,Ciii)). These data are also supported by the corresponding weights in the table in Figure 4(Dii). They reveal that different enzymes from a group of enzymes with presumably similar NAD(P)H binding sites are active in nuclei as compared to cytoplasm. G6PDH was identified as the mainly predominant active enzyme in the nuclei, the probabilities of enzymatic activity of LDH, SHD if bound to NADH and of GAPDH are at similar levels, due to the high similarity of the phasors of the enzymes. We expected to find CTBP1 as a NAD(P)H-dependent enzyme in the nucleus [57], but only found evidence for the presence of LDH, G6PDH, GAPDH, and SDH in there. As these are cytosolic enzymes, we expect the yet uncharacterized nuclear enzymes to have a similar fluorescence lifetime. In the cytoplasm, we found a high predominance of PDH, but also of iNOS and ADH. The contribution of CTBP1 (having a phasor identical to that of PDH) is rather improbable, as this is to the best of our knowledge mainly located in the cellular nucleus [57].

## 3. Discussion

Cellular metabolism has a strong impact on cell functions—this holds true for the majority of cell types, across species. In immunology, the need for appropriate methods for the analysis of metabolism has increased since it became evident that there is a strong cross-link between signaling pathways and the cellular metabolism, beyond the mere supply of energy. For example, the activation of B and T lymphocytes coincides with metabolic reprogramming towards a preferential usage of glycolysis [1,65,66], whereas their transition into memory cells goes along with an increase in oxidative phosphorylation. In the bone marrow, the birth place of immune cells and the site of immunological memory, various immune subsets find special microenvironments, which secure their survival and support their function. These special microenvironments are denoted as survival niches. The stable components of these niches are built by mesenchymal stromal cells (MSC), supported by the dense marrow vasculature [67,68]. Except for providing nutrients to diverse immune cell subsets, MSC may differentiate under certain conditions, e.g., during bone growth or bone regeneration after injury, into osteoblasts or chondrocytes. Under other conditions, e.g., in aged individuals or due to metabolic syndrome, they may differentiate into adipocytes [63]. Although these differentiation pathways are possible, the microenvironmental constraints which favor one or the other pathway are not yet fully understood. By using NAD(P)H-FLIM, others have provided evidence that cellular metabolism and differentiation stage of MSC are closely linked [21,42,47]. The interpretation of the NAD(P)H-FLIM data in these studies is hampered by either strongly simplified or too complex models of NAD(P)H fluorescence lifetime. If a monoexponential model is assumed, the decision whether a shorter mean fluorescence lifetime is caused by a lower NAD(P)H consumption (more free NAD(P)H) or by a higher activity of MDH or LDH cannot be made. Biexponential and multiexponential models, which facilitate this decision, are numerically instable and do not take into account the enzymatic landscape within the cell. The phasor approach proposed by Enrico Gratton and coworkers is a model-free analysis approach that circumvents the majority of numerical artifacts [69]. Nevertheless, data interpretation without a reference system of pure, relevant enzymes is also in this case not reliable.

To establish the systematic framework for a generally valid label-free NAD(P)H-FLIM data analysis, we first identified the most relevant, i.e., abundant NADH- and NADPH-dependent enzymes. These are responsible as catalyzers of biochemical reactions within the cell engine for energy supply, reductive biosynthesis, and other vital cell functions such as oxidative burst during phagocytosis. We measured the fluorescence decay of the coenzymes specifically bound to the respective enzymes, completing and validating published data from our and other laboratories. Although our reference NAD(P)H-dependent enzyme system for NAD(P)H-FLIM data is generally valid, an extension with other NAD(P)H-dependent enzymes relevant to specific cell populations or in certain tissue, organs, or pathologies is easily possible and desirable. Our systematic analysis framework of NAD(P)H-FLIM data relies on the following steps. (i) Calculating the SNR of the fluorescence intensity image; (ii) performing the phasor analysis of the time-domain NAD(P)H-FLIM data and identifying the pixel containing only free NAD(P)H; (iii) based on our reference enzyme system, calculating the enzymatic activity probability for all considered enzymes and assigning the predominantly activated enzyme to each pixel; and (iv) calculating the fraction of free to enzyme-bound NAD(P)H, as an index of metabolic activity. 

We first validated our systematic analysis framework of NAD(P)H-FLIM data on homogeneous mixtures of NAD(P)H and pure enzymes. These data revealed the fact that the SNR value has a strong impact on the quality of the phasor data and their interpretation. If focusing only on the accuracy of the phase vector averaged over an image representing the central position of the phasor cloud, our results indicate that up to SNR 5, a linear correction of its modulation length, i.e., vector length, is necessary while the phasor angle does not need any correction. Above a SNR value of 5, both length and angle of the phase vector averaged over the whole image are numerically reliable and the effects on the position of the phasor cloud are caused by biologically relevant phenomena. Therefore, although it is often mentioned that FLIM is independent of intensity—time and not intensity is the imaging criterion—this holds true only for higher SNR values (above 5) for which the decay curve properties remain unchanged. Additionally, the SNR value in an image also strongly influences the width of the phasor cloud. The width of the phasor cloud has an immediate impact on the pixel-based calculation of enzymatic activity probabilities, enzyme activity assignment, and on the fraction free to enzyme-bound NAD(P)H. Our FLIM data on pure NADH solution and on NADH:MDH mixtures revealed that for high accuracy above 97%, SNR values above 20 are necessary. These results are in line with our finding concerning the interdependency of GFP fluorescence lifetimes on the SNR values [9]. Our data on the NADH:PDH mixture revealed that lower SNR values and high similarity of the phase vectors of several enzymes, in this case of PDH as compared to CTBP1, iNOS, and ADH, lead to a lower accuracy of enzyme assignment. Although the reference enzyme system cannot be changed, the SNR values in an image can be increased by increasing fluorophore concentration or the excitation power, smoothing the raw data in time or in space, or increasing the image acquisition time; whereas, in cells, a concentration increase is not possible, as this would change the cell physiology, all the other strategies to improve the SNR are linked to experimental drawbacks. An increase of excitation power may lead to photodamage [64], spatial smoothing of the data leads to loss of subcellular information, whereas temporal smoothing of the time-domain data decreases the accuracy of fluorescence decay curves and, thus, the accuracy of the phasor analysis. Increasing the acquisition time per image lowers the repetition rate of time-lapse imaging. In living cells and tissues, fast changes may be overseen in this way. Therefore, a combination of these procedures to improve SNR is required if FLIM is to be performed in living cells or tissues.

Taking this into account, we applied our systematic analysis framework to NAD(P)H-FLIM data on stromal-like 3T3-L1 cells, which develop into adipocytes upon stimulation. We decided to increase the SNR to highly reliable values by increasing the acquisition time of the imaged cells, because these cells do not migrate. In this way, we found shorter mean NAD(P)H fluorescence lifetimes in the nuclei as compared to the cytoplasm, in line with previous results in differentiating myoblast cells [19]. However, we found no differences in metabolism at the subcellular level, but a different predominant enzymatic activity in nuclei as compared to the surrounding cytoplasm, leading to this change in the NAD(P)H fluorescence lifetime. Although G6PDH was identified as the predominant active enzyme in the nuclei, the probabilities of enzymatic activity of LDH, SHD if bound to NADH, and GAPDH are at similar levels, due to the high similarity of the phase vectors of the enzymes. We expected to find CTBP1 as a NAD(P)H-dependent enzyme in the nucleus [57], but did not find any hints for the presence of LDH, G6PDH, GAPDH, and SDH there. In the cytoplasm, we found a high predominance of PDH, but also of iNOS and ADH. The contribution of CTBP1 (having a phase vector identical to that of PDH) is rather improbable, as this is mainly located in the cellular nucleus.

Our unexpected results regarding the metabolic activity in the nuclei of stromal-like cells led us to the conviction that our systematic analysis framework of NAD(P)H-FLIM retains the potential of detecting yet unknown enzymatic mechanisms related to cellular metabolism. This will have a tremendous impact on the way we will interpret the impact of cellular metabolism of immune and stromal cell populations, at a subcellular level and under in vivo conditions [61].

## 4. Material and Methods

### 4.1. Two-Photon Microscope Setup Adequate for FLIM

Two-photon fluorescence imaging experiments were performed as previously described [33], using a specialized laser-scanning microscope based on a commercial scan head (TriMScope II, LaVision BioTec, Bielefeld, Germany). A near-infrared laser (Ti:Sa, Chameleon Ultra II, Coherent, Duisburg, Germany) tuned at 760 nm, repetition rate 80 MHz, and pulse width 140 fs was used as excitation source. The linearly polarized Ti:Sa beam was scanned over the sample by two galvanometric mirrors. A water-immersion objective lens (20×, NA 1.05, Apochromat, Olympus, Hamburg Germany) was used to focus the laser beam into the sample. The laser power was controlled by combinations of λ/2 wave-plates and polarizers. The ultrashort pulses of the laser were compressed using external compressor. NADH and NADPH fluorescence was collected in the backward direction using a dichroic mirror (775, Chroma, Marlborough, MA, USA), passed through an interference filter (466 ± 30 nm) and was detected by a GaAsP PMT (Hamamatsu, Herrsching, Germany) connected to previously described TCSPC electronics (LaVision BioTec). The TCSPC data were collected at a time resolution of 55 ps, over at least 9 ns and with a Gaussian-shaped instrument response function of 250 ps FWHM (Figure S6). In all imaging experiments, we used an average maximum laser power of 10 mW to avoid photodamage. The acquisition time for an image with a field-of-view of 200 µm × 200 µm and a digital resolution of 512 × 512 pixel was 472 ms.

### 4.2. Phasor Analysis of Time-Domain NAD(P)H-FLIM Data

Fluorescence lifetime data were measured and analyzed as previously described [33,60,69]. The phasor approach transforms the time-domain data (the fluorescence decay curve), to a virtual, normalized phase domain by calculating the discrete Fourier transformation numerically (modulations frequency = 80 MHz). The transformation leads to a complex number the real and imaginary parts of which give the coordinates of the vector in the phase domain (“phasor”). That vector originated in (0|0) and points towards the half circle (centrum at (0.5|0), radius = 0.5), due to the exponential nature of the original time domain data. Because of the value normalization, the real part of the phasor reaches from 0 to 1, and those of the imaginary part from 0 to 0.5. In this way, short fluorescence lifetimes of homogeneous fluorophores (monoexponential decay) are located on the half circle at large real values, whereas with increasing lifetime, the real value decreases.

### 4.3. Enzyme and NAD(P)H Solutions

NADH (10107735001, Roche, Basel, Switzerland), NADPH (N7505, Sigma, Darmstadt, Germany), and all enzymes were solved in 100 mM MOPS buffer (pH 7.8) (6979.4, Carl Roth, Karlsruhe, Germany). The enzymes PDH (8646-DH-050, R&D Systems, Minneapolis, MN, USA), CTBP1 (PRO-796, ProSpec, Rehovot, Israel), IDH (I5036, Sigma), GAPDH (G2267, Sigma), G6PDH (10127655001, Roche), LDH (LLDH-RO, Roche), HADH (ENZ-499, ProSpec), and MDH (LMDH-RO, Roche) were incubated in varying concentrations with NADH or NADPH, respectively. For the inhibition of LDH, the small molecule FX11 (427218, Sigma) was solved in DMSO and used in varying concentrations (10 µM, 0.5 mM, and 1 mM). All measurements were performed at room temperature.

### 4.4. T3-L1 Cell Culture and Preparation for Imaging

The culture of 3T3-L1 cells was performed in T75 cell culture flasks (658 175, Greiner, Frickenhausen, Germany) in growth medium (90% DMEM, high glucose (61965-026, Fisher Scientific GmbH, Darmstadt, Germany), 10% fetal calf serum (FCS) (S1810-500, Biowest, Nuaille, France), 1% penicillin–streptomycin (15140-122, Life Technologies GmbH, Darmstadt, Germany), and 1 mM sodium pyruvate (11360-070, Fisher Scientific GmbH)). Cells were split in a 1:10 ratio every 2 to 3 days. The utilized cultured cells were in passages 14–18.

For NAD(P)H-FLIM measurements, 240,000 cells per well were seeded into 6-well plates (657160, Greiner) in regular growth medium. The growth medium was exchanged daily. After 3 days, the medium was aspirated and replenished with imaging medium (90% DMEM, high glucose, HEPES, no phenol red (21063-029, Fisher Scientific GmbH), 10% FCS (S1810-500, Biowest)). During imaging cells were kept on 37 °C.

## Supplementary Materials

The following are available online at https://www.mdpi.com/1422-0067/20/22/5565/s1.

## References

[1] Jellusova J., Rickert R.C. (2017). A Brake for B Cell Proliferation: Appropriate responses to metabolic stress are crucial to maintain B cell viability and prevent malignant outgrowth. Bioessays.

[2] Caputa G., Castoldi A., Pearce E.J. (2019). Metabolic adaptations of tissue-resident immune cells. Nat. Immunol..

[3] Jellusova J., Cato M.H., Apgar J.R., Ramezani-Rad P., Leung C.R., Chen C., Richardson A.D., Conner E.M., Benschop R.J., Woodgett J.R. (2017). Gsk3 is a metabolic checkpoint regulator in B cells. Nat. Immunol..

[4] Huntosova V., Gay S., Nowak-Sliwinska P.M., Rajendran S.K., Zellweger M., Van Den Bergh H., Wagnières G. (2014). In vivo measurement of tissue oxygenation by time-resolved luminescence spectroscopy: Advantageous properties of dichlorotris (1, 10-phenanthroline)-ruthenium (II) hydrate. J. Biomed. Opt..

[5] Papkovsky D.B., Dmitriev R.I. (2013). Biological detection by optical oxygen sensing. Chem. Soc. Rev..

[6] Lakowicz J.R., Szmacinski H., Nowaczyk K., Johnson M.L. (1992). Fluorescence lifetime imaging of free and protein-bound NADH. Proc. Natl. Acad. Sci. USA.

[7] Denk W., Strickler J.H., Webb W.W. (1990). Two-photon laser scanning fluorescence microscopy. Science.

[8] Mossakowski A.A., Pohlan J., Bremer D., Lindquist R., Millward J.M., Bock M., Pollok K., Mothes R., Viohl L., Radbruch M. (2015). Tracking CNS and systemic sources of oxidative stress during the course of chronic neuroinflammation. Acta Neuropathol..

[9] Radbruch H., Bremer D., Guenther R., Cseresnyes Z., Lindquist R., Hauser A.E., Niesner R. (2016). Ongoing Oxidative Stress Causes Subclinical Neuronal Dysfunction in the Recovery Phase of EAE. Front. Immunol..

[10] Radbruch H., Bremer D., Mothes R., Gunther R., Rinnenthal J.L., Pohlan J., Ulbricht C., Hauser A.E., Niesner R. (2015). Intravital FRET: Probing Cellular and Tissue Function in Vivo. Int. J. Mol. Sci..

[11] Rinnenthal J.L., Bornchen C., Radbruch H., Andresen V., Mossakowski A., Siffrin V., Seelemann T., Spiecker H., Moll I., Herz J. (2013). Parallelized TCSPC for dynamic intravital fluorescence lifetime imaging: Quantifying neuronal dysfunction in neuroinflammation. PLoS ONE.

[12] Rakymzhan A., Radbruch H., Niesner R.A. (2017). Quantitative Imaging of Ca(2+) by 3D-FLIM in Live Tissues. Adv. Exp. Med. Biol..

[13] Radbruch H., Mothes R., Bremer D., Seifert S., Kohler R., Pohlan J., Ostendorf L., Gunther R., Leben R., Stenzel W. (2017). Analyzing Nicotinamide Adenine Dinucleotide Phosphate Oxidase Activation in Aging and Vascular Amyloid Pathology. Front. Immunol..

[14] Bayerl S.H., Niesner R., Cseresnyes Z., Radbruch H., Pohlan J., Brandenburg S., Czabanka M.A., Vajkoczy P. (2016). Time lapse in vivo microscopy reveals distinct dynamics of microglia-tumor environment interactions-a new role for the tumor perivascular space as highway for trafficking microglia. Glia.

[15] Tomkova S., Misuth M., Lenkavska L., Miskovsky P., Huntosova V. (2018). In vitro identification of mitochondrial oxidative stress production by time-resolved fluorescence imaging of glioma cells. Biochim. Biophys. Acta.

[16] Kumar S., Dunsby C., De Beule P.A., Owen D.M., Anand U., Lanigan P.M., Benninger R.K., Davis D.M., Neil M.A., Anand P. (2007). Multifocal multiphoton excitation and time correlated single photon counting detection for 3-D fluorescence lifetime imaging. Opt. Express.

[17] Stringari C., Cinquin A., Cinquin O., Digman M.A., Donovan P.J., Gratton E. (2011). Phasor approach to fluorescence lifetime microscopy distinguishes different metabolic states of germ cells in a live tissue. Proc. Natl. Acad. Sci. USA.

[18] Stringari C., Nourse J.L., Flanagan L.A., Gratton E. (2012). Phasor fluorescence lifetime microscopy of free and protein-bound NADH reveals neural stem cell differentiation potential. PLoS ONE.

[19] Wright B.K., Andrews L.M., Jones M.R., Stringari C., Digman M.A., Gratton E. (2012). Phasor-FLIM analysis of NADH distribution and localization in the nucleus of live progenitor myoblast cells. Microsc. Res. Tech..

[20] Wright B.K., Andrews L.M., Markham J., Jones M.R., Stringari C., Digman M.A., Gratton E. (2012). NADH distribution in live progenitor stem cells by phasor-fluorescence lifetime image microscopy. Biophys. J..

[21] Guo H.W., Chen C.T., Wei Y.H., Lee O.K., Gukassyan V., Kao F.J., Wang H.W. (2008). Reduced nicotinamide adenine dinucleotide fluorescence lifetime separates human mesenchymal stem cells from differentiated progenies. J. Biomed. Opt..

[22] Gratton E., Breusegem S., Sutin J., Ruan Q., Barry N. (2003). Fluorescence lifetime imaging for the two-photon microscope: Time-domain and frequency-domain methods. J. Biomed. Opt..

[23] Elson D., Requejo-Isidro J., Munro I., Reavell F., Siegel J., Suhling K., Tadrous P., Benninger R., Lanigan P., McGinty J. (2004). Time-domain fluorescence lifetime imaging applied to biological tissue. Photochem. Photobiol. Sci..

[24] Soloviev V.Y., Tahir K.B., McGinty J., Elson D.S., Neil M.A., French P.M., Arridge S.R. (2007). Fluorescence lifetime imaging by using time-gated data acquisition. Appl. Opt..

[25] Suhling K., French P.M., Phillips D. (2005). Time-resolved fluorescence microscopy. Photochem. Photobiol. Sci..

[26] Poland S.P., Krstajic N., Monypenny J., Coelho S., Tyndall D., Walker R.J., Devauges V., Richardson J., Dutton N., Barber P. (2015). A high speed multifocal multiphoton fluorescence lifetime imaging microscope for live-cell FRET imaging. Biomed. Opt. Express.

[27] Agronskaia A.V., Tertoolen L., Gerritsen H.C. (2004). Fast fluorescence lifetime imaging of calcium in living cells. J. Biomed. Opt..

[28] Gerritsen H.C., Asselbergs M.A., Agronskaia A.V., Van Sark W.G. (2002). Fluorescence lifetime imaging in scanning microscopes: Acquisition speed, photon economy and lifetime resolution. J. Microsc..

[29] Scott T.G., Spencer R.D., Leonard N.J., Weber G. (1970). Synthetic spectroscopic models related to coenzymes and base pairs. V. Emission properties of NADH. Studies of fluorescence lifetimes and quantum efficiencies of NADH, AcPyADH,[reduced acetylpyridineadenine dinucleotide] and simplified synthetic models. J. Am. Chem. Soc..

[30] Lakowicz J.R., Szmacinski H., Nowaczyk K., Berndt K.W., Johnson M. (1992). Fluorescence lifetime imaging. Anal. Biochem..

[31] Squire A., Verveer P.J., Bastiaens P.I. (2000). Multiple frequency fluorescence lifetime imaging microscopy. J. Microsc..

[32] Verveer P.J., Rocks O., Harpur A.G., Bastiaens P.I. (2006). FLIM measurements and frequency domain FLIM data analysis. CSH Protoc..

[33] Leben R., Ostendorf L., van Koppen S., Rakhymzhan A., Hauser A.E., Radbruch H., Niesner R.A. (2018). Phasor-Based Endogenous NAD(P)H Fluorescence Lifetime Imaging Unravels Specific Enzymatic Activity of Neutrophil Granulocytes Preceding NETosis. Int. J. Mol. Sci..

[34] Chance B. (2004). Mitochondrial NADH redox state, monitoring discovery and deployment in tissue. Methods Enzymol.

[35] Chacko J.V., Eliceiri K.W. (2019). Autofluorescence lifetime imaging of cellular metabolism: Sensitivity toward cell density, pH, intracellular, and intercellular heterogeneity. Cytometry A.

[36] Xu H.N., Tchou J., Chance B., Li L.Z. (2013). Imaging the redox states of human breast cancer core biopsies. Adv. Exp. Med. Biol..

[37] Bird D.K., Yan L., Vrotsos K.M., Eliceiri K.W., Vaughan E.M., Keely P.J., White J.G., Ramanujam N. (2005). Metabolic mapping of MCF10A human breast cells via multiphoton fluorescence lifetime imaging of the coenzyme NADH. Cancer Res..

[38] Skala M.C., Riching K.M., Bird D.K., Gendron-Fitzpatrick A., Eickhoff J., Eliceiri K.W., Keely P.J., Ramanujam N. (2007). In vivo multiphoton fluorescence lifetime imaging of protein-bound and free nicotinamide adenine dinucleotide in normal and precancerous epithelia. J. Biomed. Opt..

[39] Skala M.C., Riching K.M., Gendron-Fitzpatrick A., Eickhoff J., Eliceiri K.W., White J.G., Ramanujam N. (2007). In vivo multiphoton microscopy of NADH and FAD redox states, fluorescence lifetimes, and cellular morphology in precancerous epithelia. Proc. Natl. Acad. Sci. USA.

[40] Provenzano P.P., Eliceiri K.W., Keely P.J. (2009). Multiphoton microscopy and fluorescence lifetime imaging microscopy (FLIM) to monitor metastasis and the tumor microenvironment. Clin. Exp. Metastasis.

[41] Meleshina A.V., Cherkasova E.I., Shirmanova M.V., Klementieva N.V., Kiseleva E.V., Snopova Lcapital Ve C., Prodanets N.N., Zagaynova E.V. (2015). Influence of mesenchymal stem cells on metastasis development in mice in vivo. Stem Cell Res. Ther..

[42] Meleshina A.V., Dudenkova V.V., Bystrova A.S., Kuznetsova D.S., Shirmanova M.V., Zagaynova E.V. (2017). Two-photon FLIM of NAD(P)H and FAD in mesenchymal stem cells undergoing either osteogenic or chondrogenic differentiation. Stem Cell Res. Ther..

[43] Meleshina A.V., Dudenkova V.V., Shirmanova M.V., Shcheslavskiy V.I., Becker W., Bystrova A.S., Cherkasova E.I., Zagaynova E.V. (2016). Probing metabolic states of differentiating stem cells using two-photon FLIM. Sci. Rep..

[44] Evers M., Salma N., Osseiran S., Casper M., Birngruber R., Evans C.L., Manstein D. (2018). Enhanced quantification of metabolic activity for individual adipocytes by label-free FLIM. Sci. Rep..

[45] Lindquist R.L., Bayat-Sarmadi J., Leben R., Niesner R., Hauser A.E. (2018). NAD(P)H Oxidase Activity in the Small Intestine Is Predominantly Found in Enterocytes, Not Professional Phagocytes. Int. J. Mol. Sci..

[46] Alturkistany F., Nichani K., Houston K.D., Houston J.P. (2019). Fluorescence lifetime shifts of NAD(P)H during apoptosis measured by time-resolved flow cytometry. Cytometry A.

[47] Sharick J.T., Favreau P.F., Gillette A.A., Sdao S.M., Merrins M.J., Skala M.C. (2018). Protein-bound NAD(P)H Lifetime is Sensitive to Multiple Fates of Glucose Carbon. Sci. Rep..

[48] Niesner R., Peker B., Schlusche P., Gericke K.H. (2004). Noniterative biexponential fluorescence lifetime imaging in the investigation of cellular metabolism by means of NAD(P)H autofluorescence. Chemphyschem.

[49] Strickler S.J., Robert A.B. (1962). Relationship between Absorption Intensity and Fluorescence Lifetime of Molecules. J. Chem. Phys..

[50] Behne M.J., Meyer J.W., Hanson K.M., Barry N.P., Murata S., Crumrine D., Clegg R.W., Gratton E., Holleran W.M., Elias P.M. (2002). NHE1 regulates the stratum corneum permeability barrier homeostasis. Microenvironment acidification assessed with fluorescence lifetime imaging. J. Biol. Chem..

[51] Niesner R., Peker B., Schlusche P., Gericke K.H., Hoffmann C., Hahne D., Muller-Goymann C. (2005). 3D-resolved investigation of the pH gradient in artificial skin constructs by means of fluorescence lifetime imaging. Pharm. Res..

[52] Celli A., Sanchez S., Behne M., Hazlett T., Gratton E., Mauro T. (2010). The epidermal Ca(2+) gradient: Measurement using the phasor representation of fluorescent lifetime imaging. Biophys. J..

[53] Blacker T.S., Mann Z.F., Gale J.E., Ziegler M., Bain A.J., Szabadkai G., Duchen M.R. (2014). Separating NADH and NADPH fluorescence in live cells and tissues using FLIM. Nat. Commun..

[54] Datta R., Alfonso-Garcia A., Cinco R., Gratton E. (2015). Fluorescence lifetime imaging of endogenous biomarker of oxidative stress. Sci. Rep..

[55] Niesner R., Narang P., Spiecker H., Andresen V., Gericke K.H., Gunzer M. (2008). Selective detection of NADPH oxidase in polymorphonuclear cells by means of NAD(P)H-based fluorescence lifetime imaging. J. Biophys..

[56] Lee K.C., Siegel J., Webb S.E., Leveque-Fort S., Cole M.J., Jones R., Dowling K., Lever M.J., French P.M. (2001). Application of the stretched exponential function to fluorescence lifetime imaging. Biophys. J..

[57] Zhang Q., Piston D.W., Goodman R.H. (2002). Regulation of corepressor function by nuclear NADH. Science.

[58] Madhukar N.S., Warmoes M.O., Locasale J.W. (2015). Organization of enzyme concentration across the metabolic network in cancer cells. PLoS ONE.

[59] Babior B.M. (2004). NADPH oxidase. Curr. Opin. Immunol..

[60] Ranjit S., Malacrida L., Jameson D.M., Gratton E. (2018). Fit-free analysis of fluorescence lifetime imaging data using the phasor approach. Nat. Protoc..

[61] Lindquist R.L., Niesner R.A., Hauser A.E. (2019). In the Right Place, at the Right Time: Spatiotemporal Conditions Determining Plasma Cell Survival and Function. Front. Immunol..

[62] Itkin T., Gur-Cohen S., Spencer J.A., Schajnovitz A., Ramasamy S.K., Kusumbe A.P., Ledergor G., Jung Y., Milo I., Poulos M.G. (2016). Distinct bone marrow blood vessels differentially regulate haematopoiesis. Nature.

[63] Ambrosi T.H., Scialdone A., Graja A., Gohlke S., Jank A.M., Bocian C., Woelk L., Fan H., Logan D.W., Schurmann A. (2017). Adipocyte Accumulation in the Bone Marrow during Obesity and Aging Impairs Stem Cell-Based Hematopoietic and Bone Regeneration. Cell Stem Cell.

[64] Czechowska K., Lannigan J., Wang L., Arcidiacono J., Ashhurst T.M., Barnard R.M., Bauer S., Bispo C., Bonilla D.L., Brinkman R.R. (2019). Cyt-Geist: Current and Future Challenges in Cytometry: Reports of the CYTO 2018 Conference Workshops. Cytometry A.

[65] Jellusova J. (2018). Cross-talk between signal transduction and metabolism in B cells. Immunol. Lett..

[66] O’Neill L.A., Kishton R.J., Rathmell J. (2016). A guide to immunometabolism for immunologists. Nat. Rev. Immunol..

[67] Holzwarth K., Kohler R., Philipsen L., Tokoyoda K., Ladyhina V., Wahlby C., Niesner R.A., Hauser A.E. (2018). Multiplexed fluorescence microscopy reveals heterogeneity among stromal cells in mouse bone marrow sections. Cytometry A.

[68] Zehentmeier S., Roth K., Cseresnyes Z., Sercan O., Horn K., Niesner R.A., Chang H.D., Radbruch A., Hauser A.E. (2014). Static and dynamic components synergize to form a stable survival niche for bone marrow plasma cells. Eur J. Immunol..

[69] Digman M.A., Caiolfa V.R., Zamai M., Gratton E. (2008). The phasor approach to fluorescence lifetime imaging analysis. Biophys. J..

