# Supplementary material for: Systematic Enzyme Mapping of Cellular Metabolism by Phasor-Analyzed Label-Free NAD(P)H Fluorescence Lifetime Imaging
Source: Int J Mol Sci. 2019 Nov 7;20(22):5565. doi: 10.3390/ijms20225565 (PMC6887798; doi:10.3390/ijms20225565)
Supplement: Supplementary file 1 [file ijms-20-05565-s001.pdf]

International Journal of
Molecular Sciences

Supplementary Materials

Supplement video 1. To supplement figure 1 corresponding phasor plots.

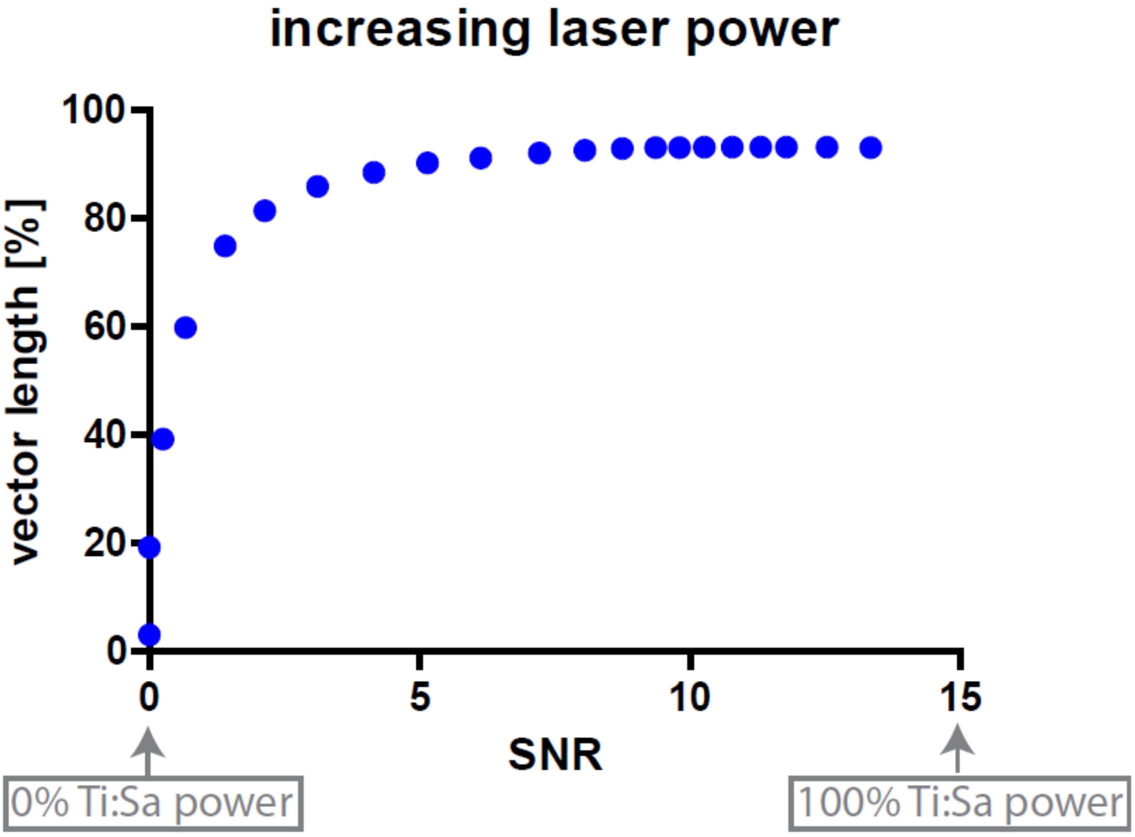

Figure S1. Free NADH solved in MOPS buffer excited with increasing laser power. Graph depicts SNR vs. vector length in percent (as described in *Results*). By increasing the excitation power, the SNR increased at a constant NADH concentration (50 µM NADH). The power at "0% Ti:Sa" is 0 mW and at "100% Ti:Sa" approx. 150 mW, it was altered in 5% steps by rotating a lambda-half plate. The SNR shows the same behavior as the increase of NADH concentration at fixed laser power (Fig. 1 Av)). In order to avoid photo bleaching and photo damage we used approx. 100 mW excitation power in the following.

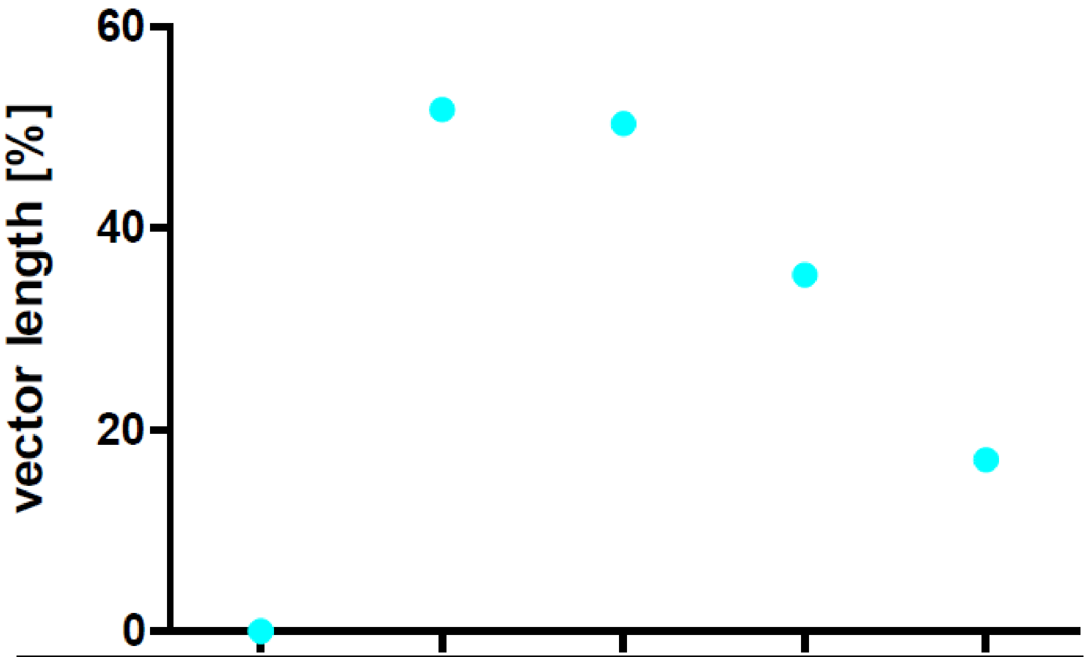

NADH	50 µM	50 µM	50 µM	50 µM	50 µM
LDH	-	10 µM	10 µM	10 µM	10 µM
FX11	-	-	10 µM	0.5 mM	1.0 mM

Figure S2. Tabled concentrations of NADH, LDH and FX11 vs. vector length. Vector length means the ratio of the vector "free NADH to the center of the center of the phasor cloud" to "free NADH to NADH fully bound to LDH position on the half circle" (cyan line in Fig. 1C), in percent. The addition of 10 µM LDH to 50 µM NADH results in an increase of the vector length ratio. The addition of FX11 to that mixture in different concentrations results in a decrease of the vector length ratio. FX11 is a LDH inhibitor, thus the phasor cloud migrates back towards free NAD(P)H with increasing FX11 concentration. (Same data as shown in Fig. 1C).

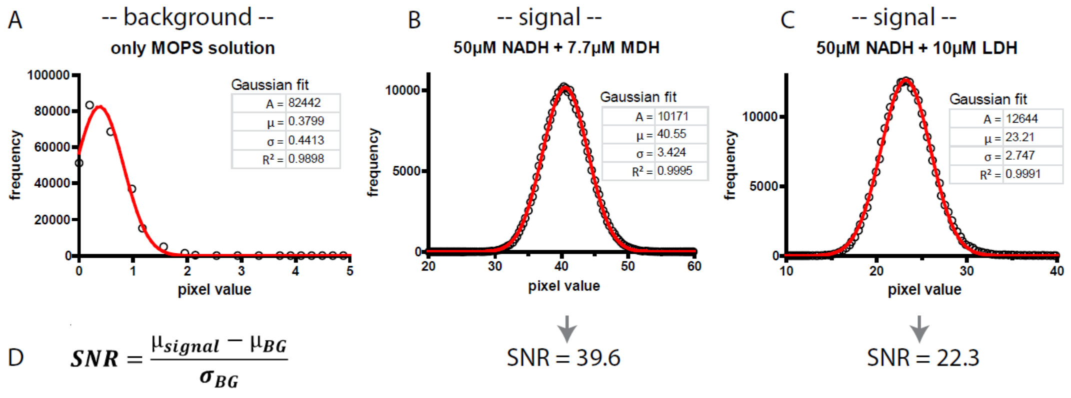

Figure 3. Calculation of signal-to-noise-ratio (SNR). **(A-C)** Gaussian fitted intensity histogram of **(A)** background measured in non-fluorescent MOPS solution (same data as shown in Fig. 1A i)), **(B)** 50 µM NADH and 7.7 µM MDH solved in MOPS solution (same data as shown in Fig. 1C iii)) and **(C)** 50 µM NADH and 10 µM LDH (same data as shown in Fig. 1C iii)). **(D)** formula to calculate SNR. μ_{signal} mean intensity signal, μ_{BG} mean intensity background, σ_{BG} standard deviation background. The SNR of (B) is 39.6 and (C) 22.3.

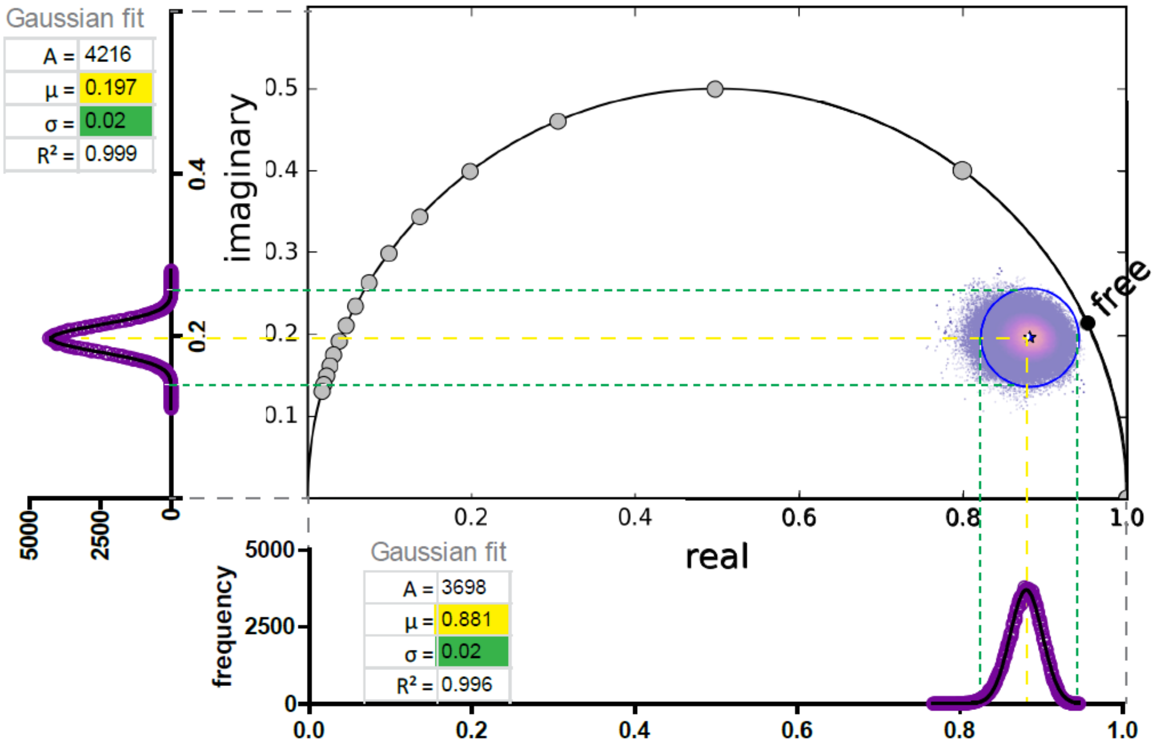

Figure S4. Determination of radius r to identify free NAD(P)H. The histograms of real and imaginary part of the dot cloud were Gaussian fitted. Used data are shown in Fig1 iv). μ_{real} and μ_{imag} give the coordinates of the center (0.881|0.197). σ_{real} and σ_{imag} are equal (σ=0.02). 3σ (=0.06) gives the radius within which, by definition, 99.7% of the values are located. Gray dots onto half circle mark $0 \leq \tau \leq 20$ ns in 1 ns steps.

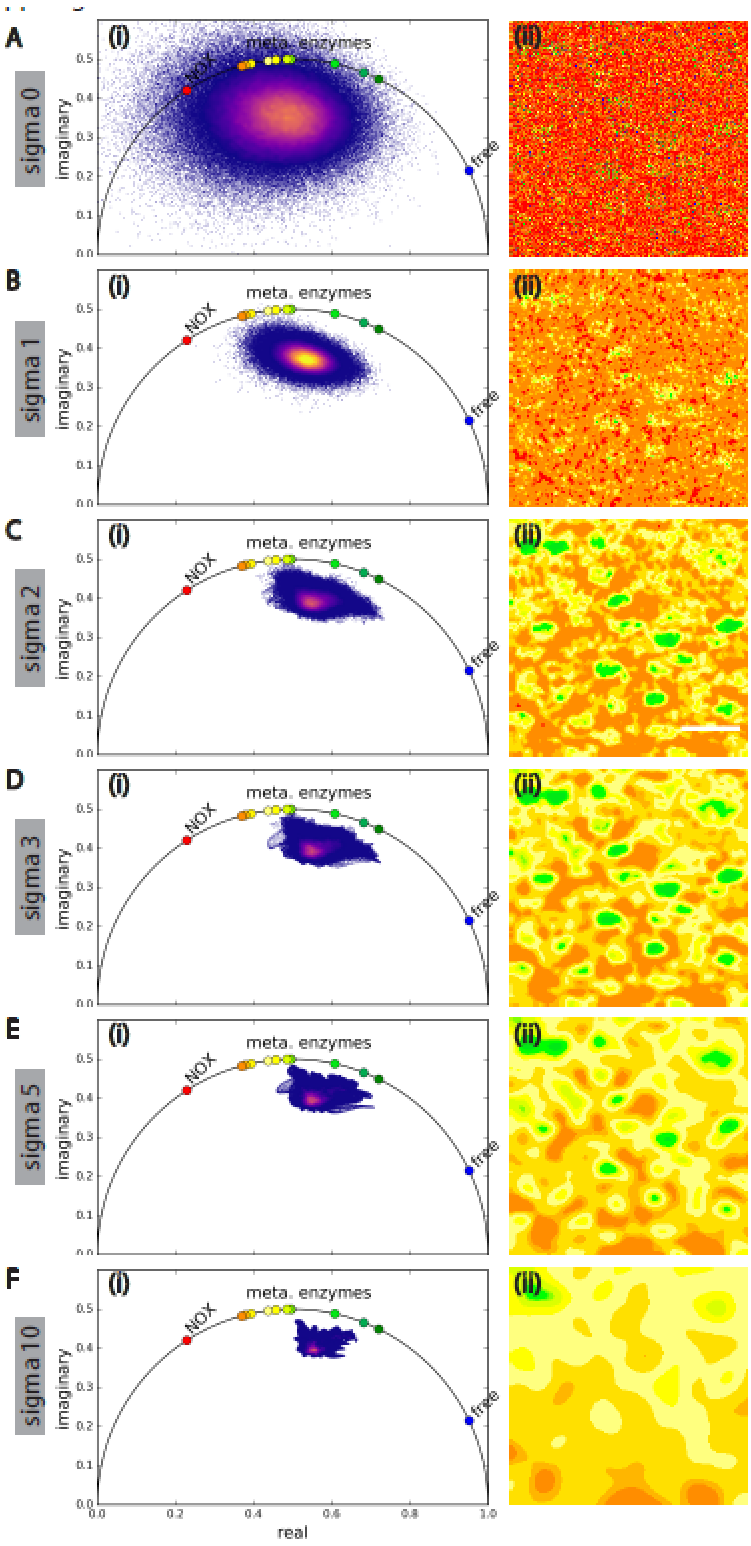

Figure S5. Influence of different sigma of Gaussian blur of time-domain raw data on phasor cloud (i) and enzyme map (ii). **A-F** same ROI as shown in Fig. 4C iii). The higher the sigma, the narrower the phasor cloud and the clearer the enzyme assignment, but at the cost of spatial resolution. If not stated otherwise, we always blurred our raw data with sigma 2 (C), which is (to our mind) a good compromise, between temporal and spatial resolution. White bar in D ii) indicates 50 μm and is valid for all enzyme maps here.

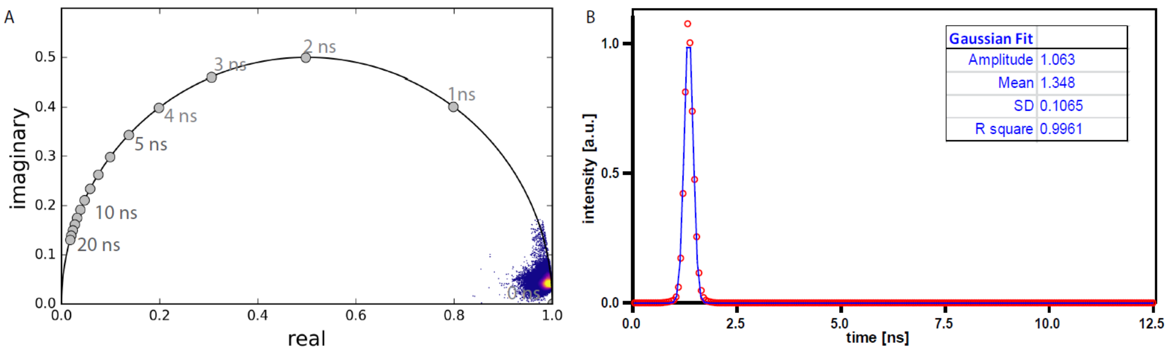

Figure S6. Instrument response function (IRF) of TCSPC measured by second harmonic generation (SHG) in potassium dihydrogen phosphate (KDP) powder (λ_{ex}=940 nm, λ_{em}=470 nm (in Ch 466/60), t_{bin}=55 ps). **(A)** phasor plot. **(B)** Gaussian fitted IRF: the standard deviation is σ=0.1065 ns, full width half maximum $FWHM = 2\sqrt{2ln2}\sigma = 250.8$ ps. The retransfer of the phase domain data into the time domain results in an average lifetime of (0.084 ± 0.015) ns, which is the smallest resolvable time of our system. The phasor cloud of the IRF lies exactly where we expected it to be, so no position corrections were made in the following.
